# The Strange Case of Peri-Implantology

**DOI:** 10.3390/dj6020009

**Published:** 2018-04-09

**Authors:** Patrick R. Schmidlin

**Affiliations:** Head Division of Periodontology and Peri-Implant Diseases, Clinic of Preventive Dentistry, Periodontology and Cariology, Center of Dental Medicine, University of Zurich, Plattenstrasse 11, 8032 Zurich, Switzerland; patrick.schmidlin@zzm.uzh.ch

Oral implantology can be considered—without any doubt—as one of the greatest achievements in dental medicine over the last hundred years. The use of this technology allows for the fixed rehabilitation of virtually any lost tooth, especially in conjunction with the progress made in guided bone regeneration (GBR). Finally, dentists—as a small and humble fraction of the “Gods in White” population on Earth—are able to act as such and are able to rebuild what is lost, although the will to do so sometimes seems a bit over the top (though this may be the topic of another editorial).

But every great story has its dark side, and this one has also provided some sad, and sometimes dramatic, chapters [1]. Just as every successful and heroic intervention may end up with complications, the risk of infirmity and failure is ever present. Although experts have mainly focused on describing clinical signs and explaining potential pathways regarding implantation, difficulties also exist in defining a common treatment concept, which is reflected in a the failure to name the subject so far.

The term peri-implantology may adequately describe—in analogy to periodontology (from Greek: παρά (parà) “next”, όδούς (odous) “the tooth” and λόγος (lógos) “word, doctrine”)—the doctrine of the tissue apparatus around implants in health and disease. Along this line, the main goals of peri-implantology are the prevention, treatment, and supportive peri-implant therapy around artificial roots. Okay! Implantologists and surgeons may argue that this may be in competition to their fields. However, this is not the case. As in dentistry and periodontology, implantology covers many important aspects: surgery (including case selection, treatment planning, pre-prosthetic surgery, GBR, etc.), prosthetics, and all related fields in dentistry, which are required to successfully treat implant patients. Peri-implantology is just the specific branch/topic when it comes to basic science, prevention, and treatment of peri-implant health and disease.

Peri-implantology should be implemented and taught as a specialty in a related discipline (e.g., periodontology or implantology). Despite political controversies, however, a patient-centered and patient-oriented education and research focus should lead to an academic strategy, which should be identified soon to realize this terminology and specialty in a university setting. Periodontists and implantologists should one day become peri-implantologists as well.

But in any case, we should never forget: Our patients were all born with teeth and we were primarily trained as dentists, with the main goal of preserving teeth in healthy conditions!

## References

[1] Levignac J. (1965). Periimplantation osteolysis-periimplantosis—Periimplantitis. Rev. Fr. Odontostomatol..

